# Clog-Free Trilobite Filtration: Tunable Flow Setup and Velocity Measurements

**DOI:** 10.3390/mi11100904

**Published:** 2020-09-29

**Authors:** Endre Joachim Mossige, Atle Jensen

**Affiliations:** Department of Mathematics, University of Oslo (UiO), 0851 Oslo, Norway; atlej@math.uio.no

**Keywords:** clog-free filtration, tunable flow setup, velocimetry, micro particle image velocimetry (*μ*PIV), particle tracking velocimetry (PTV)

## Abstract

The ability to separate and filter out microscopic objects lies at the core of many biomedical applications. However, a persistent problem is clogging, as biomaterials stick to the internal chip surface and limit device efficiency and liability. Here, we review an alternative technique that could solve these clogging issues. By leveraging tunable flow fields and particle inertia around special trilobite-shaped filtration units, we perform filtration of plastic beads by size and we demonstrate sorting of live cells. The separation and filtration are performed completely without signs of clogging. However, a clog-free operation relies on a controlled flow configuration to steer the particles and cells away from the filter structures. In this paper, we describe the tunable flow system for such an operation and we describe an optical setup enabling hydrodynamical interactions between particles and cells with the flow fields and direct interactions with the filter structures to be characterized. The optical setup is capable of measuring particle and flow velocities (by Particle Tracking Velocimetry (PTV), Micro Particle Image Velocimetry (μPIV), and streakline visualization) in meters per second necessary to avoid clogging. However, accurate measurements rely on strict calibration and validation procedures to be followed, and we devote a substantial portion of our paper to laying out such procedures. A comparison between μPIV data and a known flow profile is particularly valuable for assessing measurement accuracy, and this important validation has not been previously published by us. The detail level in our description of the flow configuration and optical system is sufficient to replicate the experiments. In the last part of the paper, we review an assessment of the device performance when handling rigid spheres and live cells. We deconvolute the influences of cell shape from effects of size and find that the shape has only a weak influence on device performance.

## 1. Introduction

Microfluidics [1,2,3] concerns the manipulation of fluid flows inside microfluidic geometries. The main advantage of microfluidics is arguably the ability to manipulate individual fluid streams [4], enabling a wide range of applications in micro-electromechanical system MEMS and in biomedical research, including reaction kinetics [5], controlled chemical synthesis [6], and drug screening [7]. Particularly, the precision offered by a microfluidics approach enables versatility in particle and cell separation applications [8,9,10,11]. The ability to manipulate particles in a continuous manner without applying external force fields, for example electric fields, is fundamental in sorting technologies such as deterministic lateral displacement (DLD) [12,13,14], inertial microfluidics [15], and hydrodynamic filtration [16].

**Figure 1:**
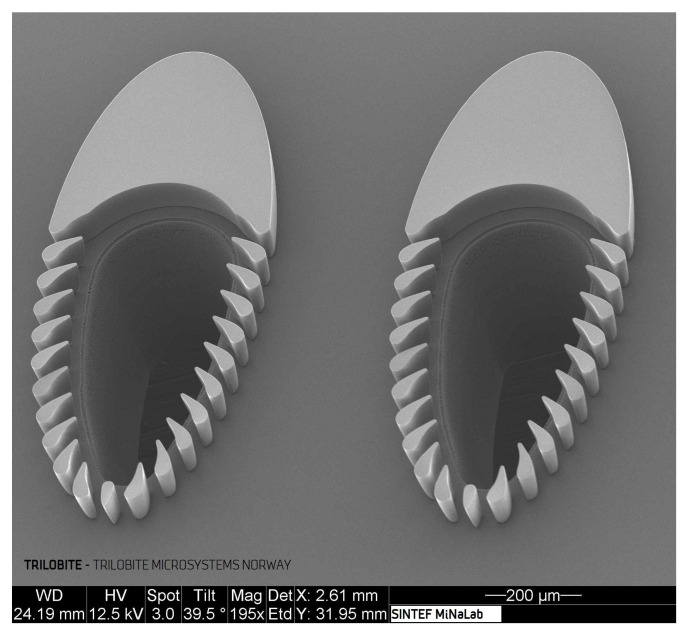
Scanning Electron Microscope (SEM) image showing two trilobite filtration units: the streamlined shape is meant to prevent clogging. The turbine blade-shaped filter blades prevent particles larger than 25 μm from entering the filtration outlets, seen as the hole in the middle of the trilobite filtration units.

This paper reviews an alternative technique named “trilobite filtration”, first described by Dong et al. [17,18]. In trilobite filtration, particles and cells are filtered by flowing around special trilobite-shaped filter units. Figure 1 is a Scanning Electron Microscope (SEM) image showing two such filtration units (1155 μm long, 460 μm wide, and center to center distance of 740 μm). The streamlined filtration posts (placed 25 μm apart) mechanically block out particles exceeding the separation gaps while simultaneously guiding filtration fluid containing small particles to the filtrate outlet, located in the center of these trilobite units. The streamlined shape of these units is meant to be clog-preventive; however, without appropriate flow control, the trilobite filter is simply a dead-end filter. This mode of operation was presented by Dong [17] for treatment of plastic spheres and, later, by Hönsvall et al. [19] for dewatering of fixed (dead) algal cells. These authors were able to concentrate spherical cells that were much larger than the filter size, but smaller cells and particles either slipped through or clogged the device.

Our efforts have focused on developing a clog-free flow configuration for the trilobite filter, enabling cells and particles over a broad size range to be handled without clogging. The flow fields used for filtration can be used to handle both synthetic materials such as microplastics and live biological material such as algal cells. By flowing the particle solutions at high speeds around the trilobite-shaped filter units, particles are directed away from the filter pores by inertial effects. Clogging is avoided by this filtration mode since it efficiently minimizes the degree of direct interaction with the filter structures. In a previous paper [20], we described how tunable flow fields and particle inertia can be utilized to mitigate the clogging issue for sized-based filtration of spherical polystyrene beads. Later, we utilized a similar flow configuration to separate live cells having various shape, size, and deformability [21]. In this paper, we focus on the experimental setup used in clog-free trilobite filtration, and the level of detail provided should be sufficient to replicate our experiments. Our micro-velocimetry setup (μPIV and PTV) is optimized for studying inertial microfluidic flows, enabling high velocity particle-wall and particle-flow (hydrodynamical) interactions, to be described. This paper also reveals new details on the optical method. Particularly, we demonstrate how to compensate for optical aberations inherent to microfluidic velocimetry, and for the first time, we present a validation of our experimental velocity measurements by comparison to an analytical flow profile. In the last part of the paper, we review the separation and filtration performance of the device.

The paper is organized as follows. The first section describes the filtration technology. The second section explains the microfluidic chip layout and the tunable flow system. The third section explains the optical system used to measure the high-velocity microfluidic flows. The fourth section explains the particles and cells used in the velocity measurements and in the filtration study. The fifth section explains the optical method used to quantify fluid and particle velocities. The sixth section reviews the performance of the device, and a conclusion summarizes the paper.

## 2. Filtration Method

**Figure 2:**
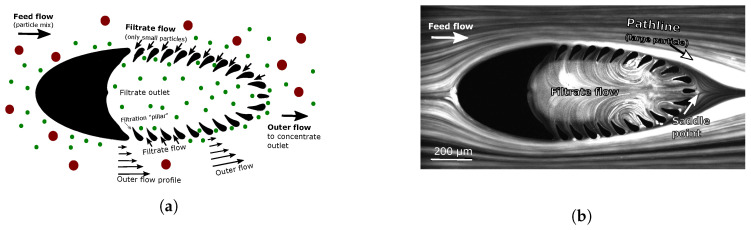
(**a**): Cartoon showing a clog-preventive flow configuration: the flow is mostly parallel to the filter blades to minimize interactions with these. (**b**): Pathline representation showing the trajectory of large separation spheres (around 30 μm) and the flow fields (obtained using 1 μm tracers) around a single filtration unit: due to particle inertia, which is achieved by pumping the fluids at high rates through the channels, the large spheres separate from the flow upstream of the so-called saddle point.

Figure 2a is a cartoon showing a clog-free operation. An incoming feed flow containing particles of various sizes is split into a filtrate and a concentrate by a trilobite filter unit. The pillars mechanically block out particles exceeding the separation gap (25 μm), allowing only small (green) particles to enter the filtrate. The excess fluid is the concentrate, and this fluid also contains particles (red) that are larger than the separation gaps. Large particles interact with the pillars; however, these interactions are minimized by the parallel flow around the trilobite units, which efficiently guides the particles away from the filter pores.

Figure 2b is a long-exposure image showing trajectories of large spherical beads (>25 μm) around a single filter unit. Due to a favorable flow field, these particles do not clog the device. The thin lines (produced by 1 μm tracer particles) represent the flow field. Note the existence of a saddle (stagnation) point directly downstream of the unit. Such low-velocity regions are prone to clogging as the velocity field is directed inwards towards the filter pores. To mitigate the clogging issue, particle inertia is utilized to defect the spheres away from the filter structures and into ambient flow.

**Figure 3:**
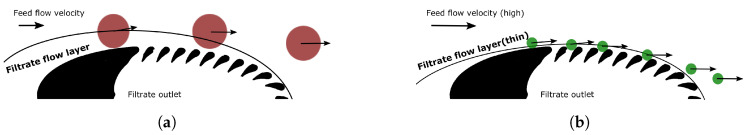
Diagram showing how tunable filtration layers around the trilobite structure are used to control the cutoff size: a particle is directed downstream to a retentate outlet by texternal flow when its center is outside of the filtrate layer. (**a**) Filtering of large spheres by a thick filtration layer and (**b**) filtration of small spheres by a thin filtration layer are shown.

The incoming particle-laden fluid that ends up as filtrate forms a flow layer around the trilobite structure; see Figure 3. The extent of this (filtrate) flow layer determines the total flow of filtrate relative to the inflow rate. For a fixed saddle point position (clog-free operation requires the saddle point to be located immediately downstream of the trilobite), the layer thickness is inversely related to the inflow rate. Particles with their center-of-mass being outside the flow layer are carried away from the filter structures by external flow, and the extent of the filtrate layer therefore dictates the particle cutoff. As a result, the maximal layer thickness ensuring clog-free operation is higher for large particles than for small ones, as shown in Figure 3. The maximal extent of the flow layer ensuring clog-free operation for filtering large (red) spheres is much thicker than the flow layer used to filter small (green) spheres. As a result, large particles can be filtered at higher concentration ratios (defined here as the particle concentration in the concentrated retentate relative to particle concentration in the incoming feed flow) than small particles. For example, the concentration ratio of 69 μm spheres was found to be almost 4 times higher than the concentration ratio of 18 μm spheres. The performance of the device for filtering particles and cells of different sizes is treated in more detail in the Results section of the paper.

**Figure 4:**
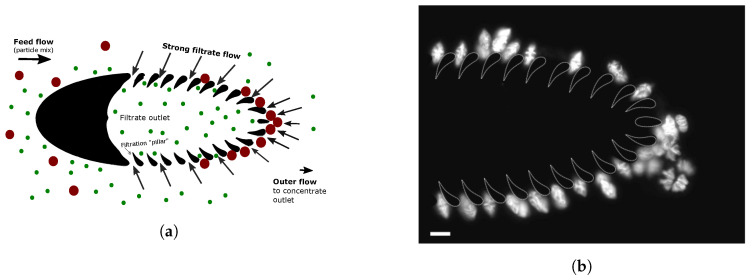
(**a**) In the absence of flow control, the filter displays dead-end filtration behavior, which is associated with clogging issue. (**b**) Clogging of a disk-shaped algal cell (37 by 77 μm) as a result of dead-end filtering. Scale bar: 50 μm.

Figure 4a is a cartoon showing an unfavorable flow configuration, which is typical in the absence of flow control. Here, a strong filtrate flow leads to dead-end filtering, with the inevitable result of clogging. Figure 4b shows clogging by the disk-like algal cell *Micrasterias truncata* (having mean thickness of 37 μm and diameter of 77 μm) as a result of dead-end filtering. However, by controlling the flow fields and by utilizing particle inertia, interactions with filter pores can be minimized to mitigate clogging issue. We have previously sorted live algal cells of various shapes (rods, spheres, and disks) and sizes (from 17 to 77 μm) by this method [21].

The filtration performance for treatment of algal cells was found to be comparable to the filtration performance for treatment of synthetic beads, even when the cells have complex shapes. This similarity in performance across different types of separation objects offers a high degree of flexibility as the device can be calibrated using well-defined spheres for real-life applications involving complex cells. The insensitivity to particle type is a convenience of utilizing hydrodynamic stresses to steer particles and cells away from the filter structures. Details on the filtration performance for handling live algal cells are provided in the Results section of the paper.

## 3. Microfluidic Chip Layout and Tunable Fluid Flow System

In this section, we describe the microfluidic chip design and the tunable flow system.

**Figure 5:**
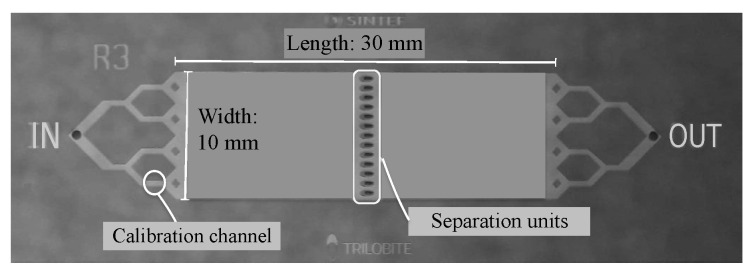
Microfluidic chip: the feed flow enters through the inlet, “IN” and flows from left to right through branching channels and a main channel (10 mm wide and 30 mm long) at a flow rate of $Q_{in}$. An array of filter units, 13 in total, serve to separate the permeate flow (flow rate $Q_{p}$) from the concentrate flow (flow rate $Q_{c}$). The concentrate exits through the downstream outlet, “OUT”, while the permeate exits through an outlet directly below the separation units. The total flow rate, $Q_{in}$, and the concentration ratio, $Q_{p}$/$Q_{c}$, are monitored by weighing the permeate and concentrate reservoirs.

Figure 5 shows the microfluidic chip layout. During an experiment, unfiltered feed fluid enters with flow rate $Q_{in}$ through the inlet (“IN”, diameter: 1 mm) on the chip and is directed to the main channel (30 mm long, 10 mm wide, and 90 μm deep) via branching channels. One of these branching channels is used for calibration purposes. Four quadratic posts prevent the fluid from degassing and serve to distribute the fluid evenly across the main channel. An array of trilobite-shaped separation units, 13 in total, is placed midway downstream the main channel, and the total flow rate of permeates through these filter units is $Q_{p}$. Each of the 13 trilobites is 1155 μm long and 460 μm wide, and the center-to-center distance between them is 740 μm. A channel network downstream of the filter units serves to guide the concentrated fluid (having flow rate $Q_{c}$) to a retentate outlet (“OUT”, diameter: 1 mm). A glass slide (300 μm thick) is bonded onto the fluidic chip for sealing and viewing purposes. The chips were manufactured using standard micromachining processes as described previously [22].

**Figure 6:**
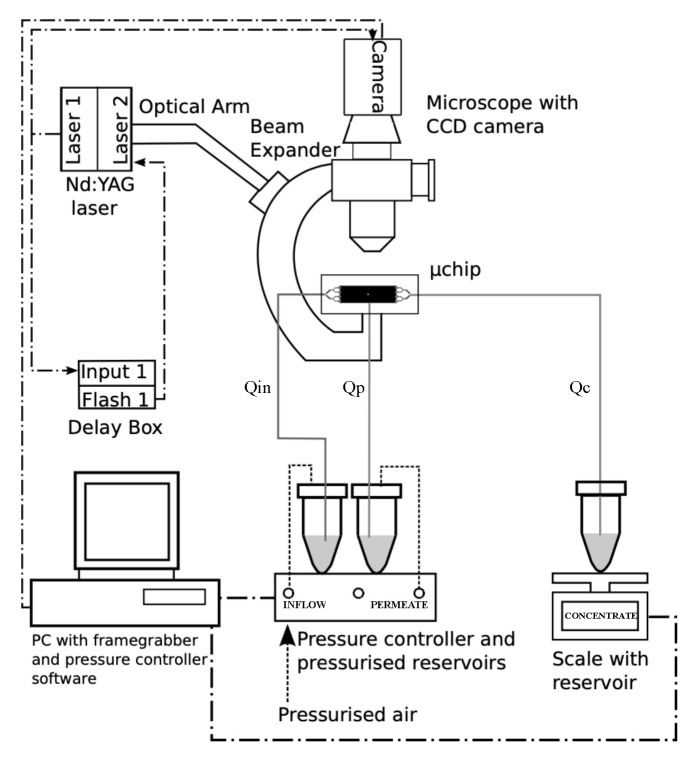
Experimental setup. Flow configuration: the “Inflow” containing particles is driven by a user-controlled pressure gradient at a flow rate of $Q_{in}$. The flow rate of permeate, $Q_{p}$, is controlled by the pressure difference between the permeate outlet and a pressurized permeate reservoir. $Q_{p}$ is determined by weighing the permeate reservoir. The unfiltered concentrate is collected in a third reservoir and a scale serves to monitor the flow rate, $Q_{c}$. In order to allow the concentrate to flow with minimal resistance, the concentrate reservoir is not pressurized. Optical system: a microscope connects to a light source (either a double-pulsed Nd:YAG laser (Micro Particle Image Velocimetry (μPIV)/Particle Tracking Velocimetry (PTV)) or a mercury lamp providing continuous luminescence (pathline visualizations and channel inspection)), and a digital camera is used for image acquisition. An optical arm guides the laser beam safely to the microscope, and a liquid light guide guides the continuous light to the microscope. The sequence of laser pulse events is controlled by two delay boxes that interface with the laser and computer via a home-built LabView program.

Figure 6 shows the experimental flow setup. $Q_{in}$ denotes the flow rate of the unfiltered fluid (“Inflow”), $Q_{p}$ denotes the filtered (“Permeate”) fluid, and $Q_{c}$ denotes the concentrated fluid (“Concentrate”). During an experiment, the lab operator controls the flow rates $Q_{in}$ and $Q_{p}$ by imposing pressures in the respective reservoirs using a computer-controlled software and pressure system (MFCS-EZ, Fluigent, Okabé, France). The pressures delivered by the pressure system provide steady flow fields, which is crucial for reliable filtration performance. Standard acrylic tubing connects the reservoirs to the chip. The small inner dimension of these tubes (0.25 mm) provides flow resistance, which further dampens fluctuations [23]. The concentrate fluid exits the microfluidic chip at a flow rate of $Q_{c}$ and is collected in an open retentate reservoir. The filtration performance is monitored by weighing the permeate and concentrated retentate reservoirs at a sampling rate of 1 Hz using laboratory scales (Mettler Toledo, Columbus, OH, USA) that interface with the computer. Clogging is detected by monitoring the flow rates; clogging of individual trilobite filter units leads to a decrease in the permeate flow, $Q_{p}$, and clogging of the main channel is identified as a decrease in concentrate flow, $Q_{c}$. However, these occurrences are highly unlikely when the controlled flow setup presented here is used.

Since the flow is driven by pressure differences, a pressure increase in the inflow reservoir leads to an increase in $Q_{in}$. If the reservoir pressure is held constant, this increase naturally leads to an increase in $Q_{p}$ as well. However, the increase in permeate fluid should be avoided as it leads to relocation of the saddle point to a downstream position, resulting in dead-end filtering (see Figure 4). To prevent this unfavorable flow configuration, pressure in the permeate reservoir is increased, providing a necessary resistance in the permeate flow branch. However, note that slowing the permeate flow results in a decrease in the filtrate flow layer, which leads to less concentrated retentate. As such, clog-free operation comes at the expense of a slight decrease in filtration performance.

To avoid bubbles from entering the microfluidic channels, the following preparation routine preceded each experiment: (1) Flush the device to get rid of air cavities: air bubbles and air columns in the tubing and chip were removed by flushing the device. This was done by applying a high-inlet pressure. The permeate outlet pressure was set to zero to create a high suction force through the permeate outlet that efficiently removed air cavities. (2) Reduce inlet pressure to obtain the desired inflow rate, $Q_{in}$, and (3) increase the permeate pressure (reduce $Q_{p}$) to move the saddle point upstream, closer to the trilobite structure.

Finally, it is virtually impossible to avoid dust particles from entering the channels. The chips must therefore be cleaned between successive experimental runs, and this was done by using a syringe to flush the channels subsequently with acetone, propanol, and de-ionized water. After the flushing procedure was completed, piranha solution (sulfuric acid to hydrogen peroxide, 3:1) was injected into the channels to dissolve dust particles. Note that piranha solution is highly corrosive and dangerous to the skin and that lab precautions must be used. A glass syringe was used for the injection since the strong piranha solution does not dissolve glass.

## 4. Optical System

Tunable flow setups enable accurate manipulation of particles in microfluidic geometries, and this functionality is especially useful in filtration and separation applications. From bulk data such as flow rate measurements, metrics of filtration performance (such as filtration ratios and even the degree of clogging) are available. However, critical information about flow velocity fields and particle interactions are not available without optical access to the microfluidic channels. Here, we describe the optical setup used to simultaneously measure both fluid and particle velocities inside the microfluidic geometry. We employ a custom-built laser luminescence system capable of handling ultrafast microfluidic flows (typically measured in meters per second rather than in μm/s). This high-speed setup enables particle-laden inertial flows to be quantified by μPIV and PTV.

Figure 6 is a schematic of the optical system, consisting of an upright microscope (Olympus BX43) that is connected to a double-pulsed Nd:YAG laser (nano L PIV, Litron Lasers) for particle luminescence. The particle images are captured with a charge-coupled device (CCD) camera (pco.4000, pco, pixel size: 9 μm), and the camera is mounted onto the microscope with an F-mount. The double cavity of the laser enables temporal resolution of only a few microseconds (typically 6 μs) given by the time difference between the two successive pulses, enabling particle displacements to be measured accurately by μPIV and PTV. The laser is most stable when operated at the maximum energy level; however, to avoid breakage of the optical components inside the microscope, the power of the optical beam must be reduced. A nice feature of the laser is its built-in attenuator (energy dump), enabling optical power of the laser beam entering the microscope to be reduced while operating at its maximum (and most stable) energy level. An optical arm (Ila laser) is used to guide the laser beam safely to the microscope. The optical arm connects to the microscope via a custom-made connector (Ila laser) having light-expanding optics, maximizing the field-of-view of the optical system.

In addition to laser luminescence, it is also possible to illuminate the fluidic channels by continuous light produced by a mercury lamp (U-HGLGPS, Olympus, Tokyo, Japan). Continuous luminescence is used for clogging inspection and for pathline visualizations. A liquid light guide (U-LLGAD, Olympus, Tokyo, Japan) is used to guide the light from the source to the microscope. Two different optical filter cube sets inside the microscope are used in the experiments. For inspection of channels and particles, a simple set of mirrors is used to direct light from the mercury lamp to the objective. However, for the velocity measurements and for the pathline visualizations, a filter cube set for green light (fluorescence) emission is used instead. The emission wavelength matches that of the particles used in the experiments to maximize the optical signal. The fluorescence filter cube consists of an excitation filter (ZET532/10×, Chroma Bellows Falls, VT, USA), a beam splitter (z532rdc, Chroma, Bellows Falls, VT, USA), and an emission filter (HQ 580/60, Chroma, Bellows Falls, VT, USA). The excitation filter produces a narrow-banded spectrum centered around the wavelength of the Nd:YAG laser 532±10 nm (green light). The beam splitter is a long-pass filter that blocks out wavelengths below 595 nm. Reflections of incident light inside the microfluidic channels can deteriorate the optical signal in the particle images. To avoid this from occurring, the long-pass emission filter blocks out wavelengths below 600 nm.

Four different objectives were utilized depending on application. A 4× objective: (Olympus Plan C N 4×/0.10) was used for inspection due to the large field-of-view, a 10× objective (Olympus PlanC N 10×/0.25) was used for pathline visualizations and for channel inspection. For the velocimetry measurements, two different objectives were used; a 20× objective (Olympus UPlanFL N 20×/0.50), and a 25× water-immersion objective (Olympus XLPlan N 25×/1.05).

## 5. Particles and Cells Used to Characterize the Filter Technology

In the experiments, we used tracer particles, filtration particles, and live micro algal cells.

Tracerparticles (1 μm, ρ = 1 g/cc, Life Technologies, Camarillo, CA, USA) were used for flow visualizations and for PIV measurements. The excitation peak (540 nm) was matched to the wavelength of the incident light (532 nm), resulting in a strong fluorescent signal.

Filtrationparticles: Spherical polystyrene beads (Cospheric LLC, Santa Barbara, CA, USA) with mean diameters of 21, 24, 32, and 69 μm were used for visualizing particle filtration and for the PTV measurements. The separation beads were coated with fluorescent dye that maked them fluorescent when illuminated by continuous green light as well as by the light (λ = 532 nm) produced by the laser. Both tracers and separation particles are neutrally buoyant in deionized water (the working fluid), and this convenience eases the predictions of the particle trajectories as gravitational forces can be neglected. Surfactant Triton-X (Sigma Aldrich) is added to the particle solutions to prevent bubble formation and particle agglomeration to the walls and coverslip surface.

Livealgalcells: The current technology has potential in algae-harvesting applications. In order to characterize this potential, filtration and size-based separation of live algal cells was performed. The filtration performance attained using cells were compared to those achieved using synthetic beads; see the Results section of this paper. To ease this comparison, we used single-celled organisms, which are ideal candidates for such a study due to their narrow size and shape distributions, low tendency of biofilm production, and ease of cultivation. For details on cultivation methods, see References [24,25].

The cells types used in the filtration experiments offer a wide variety in size and shape. Two marine and two freshwater species were used. The two marine species, *Prorocentrum minimum*(“M1”) and *Protoceratium reticulatum*(“M2”), were nearly spherical and so their shape was not expected to influence the filtration performance. M1 (13 by 17 μm) was slightly smaller than the filtration slits, while M2 (29 by 30 μm) was larger than the slit width. As such, in the absence of controlled flow fields, we expect the M1 cell to slip though the filter pores while the M2 cell is likely to clog the device.

The device performance was also validated for filtration of cells with complex shapes and variable stiffness. Two freshwater species were used for this purpose; the rod-shaped and flexible (19 μm wide and 41 μm long) *Cryptomonas rostratiformis* cell (“F1”) and the disk-shaped (rigid) *Micrasterias truncata* (“F2”). The motion of cells through microfluidic geometries are greatly affected by their shape, size, and rigidity, as these physical properties directly affect the interactions with the filter units and flow fields. The influence of these parameters on filtration performance with the current technology is described in detail in Mossige et al. [21] and reviewed in the Results section of this paper.

The M1, M2, and F1 cells are swimmers. As such, their swimming speed must be considered as it can influence filtration performance. The marine dinoflagellates (M1 and M2) have two flagella and have a helical swimming pattern. C.rostratiformis (F1) has two flagella, where the front with thin hairs drags the cell forward while erected. By long exposure photography, explained in the next section of this paper, it is possible to attain the swimming speed of the algal cells. Based on the length of the streaks produced by the swimming cells, their speed is estimated to ∼0.15 mm/s. This is negligible compared to the speed of cells in the flow field around the trilobite separation units (∼2 m/s) and thus does not alter their trajectories or the filtration performance.

Synthetic beads are density matched with water (the immersion medium). However, live algal cells are typically around 5% heavier than their immersion medium [26]. Direct comparison between cells and beads requires this density difference on the device performance to be characterized. This subtle density difference can give rise to weak inertial and buoyancy effects [27]. However, in light of the measurement error, these contributions can be ignored, making direct comparison between particles and cells feasible.

Finally, in order to undergo photosynthesis, algal cells are inherently fluorescent. As a result, they emit a strong fluorescent signal when illuminated by green light. This convenience allows studies of their trajectories by long exposure imaging to be performed, as explained in the next section of this paper. We also describe the methods used for flow and particle velocimetry, namely μPIV and PTV, respectively.

## 6. Optical Methods Used to Quantify Fluid and Particle Velocities

For the purpose of visualizing fluid and particle motion inside our microfluidic device, we applied pathline visualizations, micro particle image velocimetry (μPIV), and particle tracking velocimetry (PTV). This chapter serves as a mini-review of these methods and a description of how we implemented them to characterize the trilobite filter and its performance.

### 6.1. Pathline Visualizations

Pathline visualization is a well-known photographic technique to produce beautiful images of, e.g., traffic flow at night [28]. However, pathline visualizations can also be used to characterize the motion of particles in a fluid [29,30]. In that case, a long exposure image of particles that emit light against a dark background is captured and the pathlines produced by the particles represent their trajectories. In microfluidic applications, a strong optical signal between the particles of interest (in focus) and the background glow is attained by fluorescence illumination.

In the present study, pathline visualization serves three purposes. First, it is used to characterize the flow fields inside the microfluidic geometry. For this purpose, neutrally buoyant tracer particles that follow the flow accurately are used. Second, pathline visualization serves to characterize the hydrodynamical interactions between the flow fields and the large polystyrene beads called “filtration particles” (21, 24, 32, and 69 μm) as well as their collisions with the filter units. Third, pathline visualizations serve to characterize hydrodynamical interactions between the flow fields and live algal cells as well as to direct interactions with the filter structures.

In addition, valuable information about the nature of the flow can be extracted from long exposure images. For example, it is possible to attain the degree of steadiness in the flow; tracers in a steady flow produce sharp images, while tracers in an unsteady flow produce blurry images. Our long-exposure pathline visualizations show that the flows are steady, and this is used to our advantage in the particle image velocimetry measurements described below.

### 6.2. Micro Particle Image Velocimetry (μPIV): Method and Calibration

μPIV experiments served the purpose of characterizing flow fields around the trilobite filter units. The following paragraphs explain the μPIV method and how it was implemented in our experiments for characterizing the trilobite filtration technology.

Particle Image Velocimetry (PIV) [31,32,33,34] and μPIV [23,35] uses information about the displacement of colloidal particles to measure fluid flow velocity fields. When tracer particles that accurately follow the flow are used, the velocity u is found from the particle displacement, Δx, between two successive exposures with time lag Δt, yielding the velocity:

(1) $$u=\frac{\Delta x}{\Delta t}.$$

In order for Equation (1) to be accurate, Δt must be sufficiently small to ensure that the flow is purely translational without rotation and acceleration.

The particle displacement, Δx, is found by cross correlating (by a convolution integral) two successive particle images, image A(i,j) and image B(i,j), where i,j denote the pixel indices on the CCD chip of the camera. The output of the cross correlation step yields a spatially resolved correlation function, C(i,j). The location of the peak in the correlation function is used to extract the average particle displacement, which represents the average fluid displacement, Δx.

In order to obtain an accurate prediction of the correlation peak, a general criterion is that the domain is sufficiently seeded with particles [36]. This is easily achieved in macroscopic PIV setups, but the small field-of-view characteristic of microfluidic optical setups can make this difficult to achieve. Fortunately, there are well-documented methods to improve the signal strength of the correlation function that efficiently circumvents the issue of insufficient seeding.

There are three averaging techniques available to increase the quality of the correlation function and therefore the velocity calculation. These are called velocity averaging, image averaging, and ensemble averaging [37]. In velocity averaging, instantaneous velocity fields, $v_{i=1\cdots N}$, are calculated from each of the correlation functions. An average velocity field is then obtained by averaging over these instantaneous velocity fields, $v_{avg}$ = $avg(v_{i=1\cdots N})$. Velocity averaging is commonly used to smooth out and to remove erroneous velocity vectors in steady or time-periodic flows. In image averaging, an average particle image is created from an ensemble of images prior to computing the correlation function as a means to synthetically seed the domain.

In the last method, called ensemble averaging, an average correlation function, Cavg = $avg(C_{i=1\cdots N}))$, is calculated from an ensemble of correlation functions, and the velocity field, v, is then calculated from $C_{avg}$. In the original paper describing these methods for μPIV implementation [37], it was demonstrated that ensemble averaging has superior performance compared to the two alternative averaging techniques for steady microfluidic flows (faster convergence rates and signal-to-noise ratios). All of our velocity measurements were performed using an in-house-made ensemble average PIV code [38]. To converge the velocity fields and to minimize the number of erroneous velocity vectors, as much as 50 successive image pairs were used to compute a single correlation function. Finally, by subtracting a background image from the particle images prior to the cross-correlation step, the velocity measurements were further improved.

#### 6.2.1. Assessment of the Influence of Brownian Motion on μPIV Measurements

In order to achieve detailed descriptions of the flow field, it is essential that the tracers follow the flow accurately. However, collisions with fluid molecules can cause the seeding particles to fluctuate by Brownian motion. These fluctuations can deteriorate the PIV results as they introduce uncertainty in the determination of particle locations. The error associated with Brownian motions can be estimated as the ratio of the displacement due to Brownian fluctuation between exposures, $\Delta x_{B}$, to the displacement due to the flow, Δx = uΔt, yielding $E_{B}$ = $\frac{\Delta x_{B}}{\Delta x}$ = $\frac{1}{u}\sqrt{\frac{2D}{\Delta t}}$ [39]. Here, *u* is a typical flow velocity and Δt is the time shift between laser pulses. The Brownian diffusion coefficient is calculated by the Stokes–Einstein relation, $D=\frac{\kappa T}{3\pi \mu d}$ [40], where κ is a constant, *T* is temperature in Kelvin, μ is the fluid viscosity, and *d* is the particle diameter. By combining the expression of *D* with the expression for the error $E_{B}$, we get the following:

(2) $$E_{B}=\frac{\Delta x_{B}}{\Delta x}\propto \frac{1}{u}\sqrt{\frac{1}{d\Delta t}}$$

when the fluid viscosity and temperature are set by the experiment. The error associated with Brownian motion is therefore minimized by increasing the time between laser pulses, Δt (or time between exposures if a high speed camera is used in combination with a continuous light source); by increasing the particle size, *d*; or by increasing the flow velocity, *u*. In practice, Brownian motion may influence μPIV measurements in many typical microfluidic flows where the flow velocity is only a few μm/s. In our experiments, the high flow velocity (approaching 2 m/s in the region between trilobite filter units) effectively minimizes the influence of Brownian motion. Since Brownian fluctuations are random, the error is further reduced by employing ensemble averaging, as explained in the previous section. In our experiments, the maximal error due to Brownian motion is $E_{B}∼10^{-6}$ and we infer that such effects do not cause inaccuracies in our PIV method.

#### 6.2.2. Effects of Particles Being Out-Of-Focus on μPIV Measurements

In μPIV, the entire channel is illuminated by so-called “volume illumination”. As a result, particles out of focus also contribute to velocity measurements, and the influence of these particles can deteriorate the quality of the experiments. Therefore, it is important to characterize the error associated with these defocusing effects, and this is the focus of the current section.

**Figure 7:**
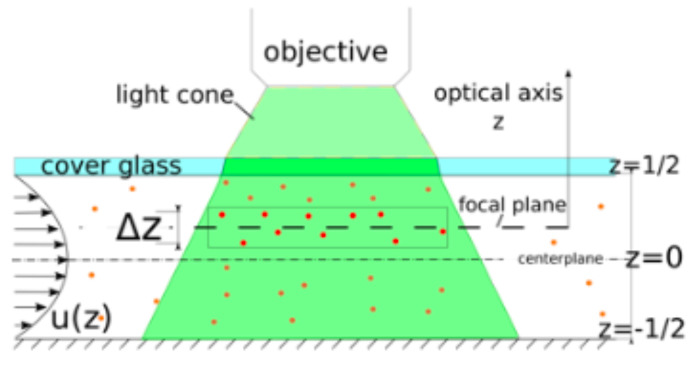
Diagram showing volume illumination of particles for μPIV measurements using an upright microscope: the true velocity profile is given by u(z), but influences from defocused particles deteriorate the velocity measurements, causing a discrepancy. For example, the measured centerplane velocity at z=0 is biased towards lower velocities. The best agreement with the true velocity profile is obtained 1/6 channel depths away from the centerplane.

Figure 7 is a side-view showing volume-based illumination of a microchannel used in our μPIV measurements. The plane of interest to us is the focal plane. However, particles within a slice with thickness of ±Δz from the focal plane (along the optical *z*-axis) contribute to the velocity measurement. The thickness Δz, known as the measurement depth, can be estimated theoretically, as shown by members of the Adrian group [41]. In Reference [41], Δz is assumed to be a function of experimental parameters such as particle size and depth of field of the objective, but influences from defocused particles are not considered. In two later papers by the same group [42,43], an improved formulae which accounts for the background “glow” of particles between the objective and the focal plane is presented.

To quantify the influence of particles being out of focus on the velocity measurements, experimentally obtained flow profiles are compared to a known velocity profile between two parallel plates, as presented in [44] and many other textbooks in fluid mechanics. For our purpose, we performed PIV measurements using one of the branching channels interconnecting the inlet with the main channel; see Figure 5. These branching channels are 500 μm wide, 1500 μm long, and 90 μm deep. In order to enable direct comparison with the analytical profile, the flow must be unidirectional. This is ensured by capturing images halfway downstream the channel, as far away from the inlets and outlets of the branching channels as possible. In order to determine the contribution of out-of-focus particles at different channel depths on the experimental flow profiles, our velocity measurements were performed at the centerline between the top and bottom plates as well as ±1/6 and ±2/6 channel depths away from the centerline; see Figure 7. A stepping wheel with 1 μm resolution served the purpose of advancing between the different measurement planes along the optical *z*-axis. This important validation is presented in the Results section.

### 6.3. Particle Tracking Velocimetry (PTV)

In order to characterize the hydrodynamical interactions between the flow field and “filtration particles” (see Section 5) we performed particle tracking velocimetry (PTV), enabling velocities of individual particles to be measured.

**Figure 8:**
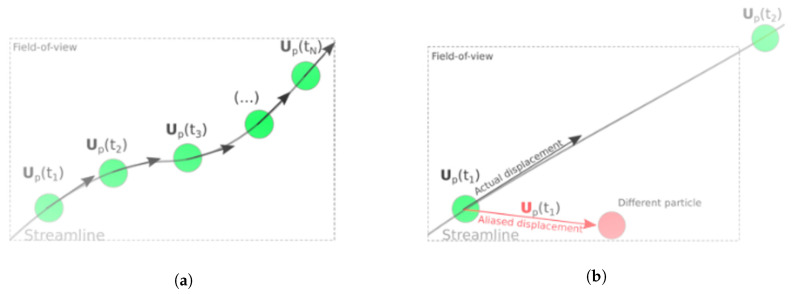
PTV measurement with (**a**) small displacement between exposures, which leads to high temporal resolution, and (**b**) large displacement between exposures (larger than the field-of-view), which leads to particle loss and erroneous velocity vector due to aliasing.

In conventional microfluidic velocimetry systems, image acquisition is attained by employing a high-speed video camera in combination with a continuous light source. These systems are suited for low-velocity microfluidic flows, where the particle displacements between exposures are typically much smaller than the field-of-view of the optical system, enabling multiple exposures of a single particle. This principle is shown in Figure 8a. However, this method is not appropriate for measuring particle velocities in inertia-based filtration devices, as in the focus of this paper. If conventional acquisition methods are applied to such flows, the displacement of a single particle can easily exceed the field-of-view (Figure 8b). The particle loss leads to aliasing if a different particle enters the field-of-view, shown here as a “different particle”, resulting in an erroneous velocity measurement. Additionally, the shutter speed of a typical high-speed camera is too slow to eliminate streaks in the particle images. False representations of the particle shapes can deteriorate the prediction of the particle locations, further degrading the velocity measurements.

To circumvent the limitations of conventional optical systems used in microfluidics, we use a double-pulsed laser in combination with a double image CCD-chip camera to acquire the particle images. The short time delay between pulses enables high particle velocities (up to 2 m/s in our experiments) to be measured accurately as no particles are lost between successive exposures. Furthermore, streaks than can deteriorate the prediction of the particle locations are completely eliminated by the short pulse duration of the laser (typically 6 ns).

A recurring challenge in microfluidic velocimetry is obtaining a sufficient number of particles in each image. For the PIV measurements, we solved this issue by employing an ensemble average PIV method. For our PTV measurements, we circumvent this issue by creating a stacked image from individual particle images before we measure their individual displacements. In laser-based luminescence, as used here, the stacked image is constructed by looping over the ensemble of images and by keeping the maximum gray scale value at each pixel location. This operation can be written as ImgMax = max(Img(i,n)), where *i* is the pixel location and *n* is the image number in the ensemble. PTV measurements are sensitive to influences from unfocused particles. In our experiments, we minimize this influence on the velocity measurements by subtracting an average background image from ImgMax before we measure the particle displacements. The background image is constructed by keeping the minimal gray scale value at each pixel location in the ensemble of particle images. This operation can be written as ImgMin = minImg(i,n) [45], and the image used in the velocity computation is ImgPTV = ImgMax − ImgMin.

## 7. Results

### 7.1. Validation of the μPIV Measurements by Comparison to an Analytical Flow Profile

**Figure 9:**
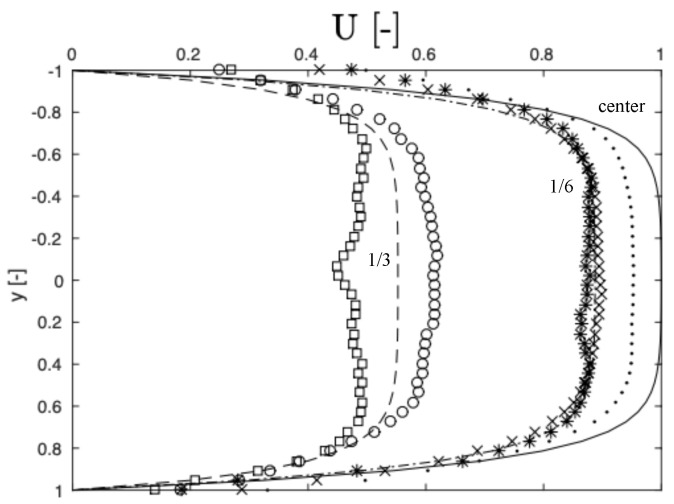
Comparison between analytical (lines) and experimental flow profiles (symbols, obtained with μPIV measurements) at different channel depths: deviations from the analytical (true) flow profile are due to aliasing effects and effects of particles being out of focus on the velocity measurement. The measured center velocity is 90% of the true velocity. The velocities (U) are normalized by the maximal in-plane velocity, and the spatial coordinate (y) is normalized by the channel width.

A comparison between experimentally obtained streamwise flow profiles and analytical flow profiles at different channel depths is presented in Figure 9. The measured profiles are shown as symbols, while the analytical solution is represented by lines. The measured profiles show excellent agreement with the analytical solution at ±1/6 channel depths away from the centerline (crosses and stars), where the two types of profile are almost indistinguishable. The reason for this excellent agreement is that positive and negative velocity contributions relative to the velocities at the focal plane cancel each other out. At the centerline, where the focal plane coincides with the centerplane of the channel, the experimental profile (dots) deviates from the analytical profile (solid line) by 5%. The reason for this deviation is that particles away from the centerplane translate at lower velocities as compared to particles at the centerplane, where the profile has its maximal value. The contribution of particles away from the focal plane to the measured velocity profile therefore leads to an underestimation.

At ±1/3 channel depths away from the centerline, the measured velocities (circles and squares) deviate strongly from the analytical profile (broken line). The experimental profiles also deviate from one another; close to the top glass slide that seals the channel, the measured velocity (circles) is higher than the analytical velocity, while the measured velocity near the bottom (boxes) is lower than the analytical velocity. The observed underprediction can, at least in part, be explained by the fact that particles adhere to the bottom, which leads to a bias towards lower velocities. The reason for this bias is that the particle images auto-correlate with themselves, as explained by Sveen and Cowen [36]. Regarding the velocity overprediction observed at +1/3 depths away from the centerplane, a possible explanation is that the measurement volume is actually partially outside of the channel (which is not improbable as this measurement plane is only 1/6 away from the top wall), meaning that the majority of particles contributing to the velocity measurements are in relatively high-velocity regions towards the centerplane.

To explain the deviation of measured velocities at different measurement planes, it can also be useful to refer to the measurement depth, Δz, as presented by Olsen and Adrian [42]. In that paper, it is stated that, by increasing the distance between the objective and the focal plane (sometimes called the penetration depth), the measurement depth, Δz, is reduced: “because when *a* [the penetration depth] is small, the bulk of the out-of-focus particles are farther from the microscope objective than when *a* is large, and thus less of their light is collected by the objective reducing the intensity of the background glow.”

Finally, spherical aberations [46] can also influence the results. As explained by Wereley and Lueptow [47], aberations cause a particle to appear elongated when viewed through a microscope. The degree of elongation increases with the penetration depth into the channel and consequently the degree of aberration is higher for particles below the centerline as compared to particles above the centerline. It is possible that this difference in deterioration of the optical signal can at least in part explain the dependency of the velocity measurements on the penetration depth. The authors of this paper are not aware of any report describing the influence of spherical aberations on the measurement depth in μPIV.

### 7.2. Velocity Measurements Around the Trilobite Filter

**Figure 10:**
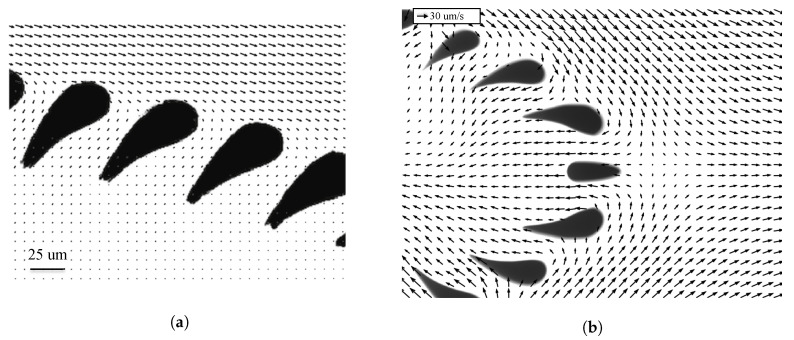
Upstream (**a**) and downstream (**b**) velocity fields around the trilobite filter unit: the flow fields were obtained using a calibrated PIV setup.

Figure 10 shows PIV velocity vectors at two different locations along a trilobite unit: (a) an upstream flow field and (b) a flow field in the downstream region surrounding the saddle point. In order to sufficiently resolve the 25 μm gap between the filtration posts, each picture was subdivided into regions spanning 46 μm by 46 μm (128 by 128 pixels) and these regions were then overlapped by 75% to improve the resolution by a factor of four, yielding a spatial resolution of 11.5 μm. An in-house-made ensemble average PIV algorithm tailored towards microfluidic flows was used to obtain the velocity fields from the particle images, and the details of this algorithm are well described in the documentation; see Reference [38].

**Figure 11:**
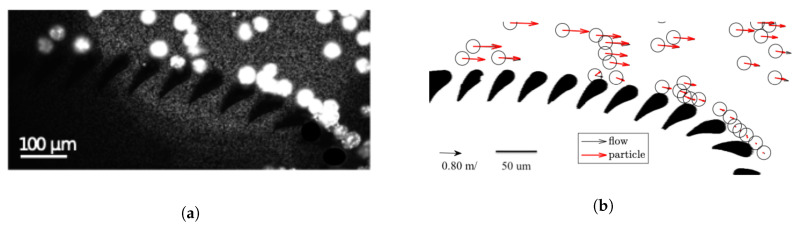
(**a**) An image constructed by stacking one hundred raw images showing filtration particles (32 μm) and tracer particles (1 μm) around a trilobite-shaped filtration unit: the background is subtracted for increased contrast. (**b**) Combined PIV (black vectors) and PTV measurement (red vectors): most of the particle and velocity vectors overlap; however, there is some tendency of particles to migrate across the flow streamlines.

In order to characterize the hydrodynamical interactions between the flow field and “filtration particles”, we performed particle tracking velocimetry (PTV). In our experiments, the velocity, $U_{p}\left(x,t\right)$, of a particle at location x is simply taken as its displacement Δx at that location during the time interval Δt. Owing to the low particle concentration, as much as one hundred particle images were used to create a single stacked image used in the PTV measurements (Figure 11a). Note that, in cases where the domain in back lit such that the particles appear dark against a bright background (as in brightfield luminescence), the particle image is constructed from the minimal intensity value at each pixel location and the background image is constructed from the maximal intensity value.

Figure 11b is an example of a PTV measurement of particle flow around a trilobite unit produced by using the particle image in Figure 11a. As can be seen from this figure, most of the particle vectors (red) and flow vectors (black) overlap; however, there is some tendency of particles to migrate across the flow streamlines. The magnitude of the so-called slip Reynolds number can be used to quantify the degree of inertia, which is believed to cause the particles to divert from the flow. The slip Reynolds number is given by $Re_{slip}=U_{slip}a/\nu$, where $U_{slip}$ is the slip velocity between a particle and the surrounding fluid, *a* is the particle diameter, and ν is the kinematic viscosity of the surrounding fluid (water). For the range of filtration particles used in the experiments, $Re_{slip}∼$ 10, which is considered high for microfluidic particle flows. The relatively high value of this parameter is a strong indicator that inertia causes the particles to drift across steamlines.

### 7.3. Filtration Performance: Synthetic Beads

In order to access the filtration performance, it is convenient to introduce the filtration ratio, *F*, defined here as the maximal achievable ratio of filtrate fluid to feed fluid. *F* is optimized by tuning the flow layer thickness, and experimentally, this is achieved by applying the smallest possible inflow rate required to separate each type of particle (21, 24, 32, and 69 μm). The filtrate flow rate is adjusted simultaneously to ensure that the saddle point is fixed near the last pillar structure, as is required to avoid clogging. The maximal filtration ratios, *F*, for each particle, 21, 24, 32, and 69 μm, are 8%, 8%, 15 %, and 29 %, respectively. The reason why large spheres can be filtered at high *F*-values is that they can roll or slide over the pillars at relatively low velocities without clogging. This mode enables thick flow layers to be used for separation. Small particles, on the other hand, cannot be filtered by rolling or sliding over the pillars as such interactions lead to clogging. Instead, they must be filtered by inertial effects to induce migration across streamlines, from the filtrate layer and into the bulk flow. The high flow rates needed to avoid clogging is associated with small filtrate flow layers, for which the thickness is inversely linked to the filtration ratio.

The 21 μm and 24 μm spheres can end up in the filtrate as they can slip though the gap separating the filtration posts (25 μm). This loss of particles leads to a reduction in the ratio of the particle concentration in the retentate to the particle concentration in the incoming feed fluid. This ratio is known as the concentration ratio, *R*, and is found to be 104%, 108%, 118%, and 142% for particles with diameters of 21, 24, 32, and 69 μm, respectively. From this, we see directly that the device performance is strongly linked to the size of the object being filtered. Using a least square fit between these data points, we found that *R* scales quadratically with particle size. The dramatic increase in *R* with particle size is also attributed to the maximal filter layer thickness allowing clog-free operation.

Based on size-exclusion arguments, the center of mass of a particle must be outside of the filtrate layer in order to avoid clogging. The reason why this particle location is advantageous is that the net hydrodynamic stress determining the particle trajectory points away from the filter blades. However, our optical measurements show that this requirement is overly strict. We have found that clog-free behavior is enabled as long as the filtrate layer is equal to the particle diameter, i.e., allowing twice as thick a filtrate layer as hypothesized based on geometric arguments. This observation is utilized to enhance the filtration performance, allowed by thick filtration layers. This performance enhancement is attributed to inertial effects, which causes particles to migrate across streamlines, from the filtrate layer and into the bulk flow.

### 7.4. Filtration Performance: Live Cells

In order to compare the filtration performance for synthetic bead filtration to the performance of the device when applied to cells, it is necessary to deconvolute effects of shape and deformability from size effects. For this purpose, it is convenient to introduce a virtual diameter, *a*, which is directly linked to the concentration ratio, *R*. By convention, *a* of a complex particle, such as the disk shaped F1 cell used in this study (19 by 41 μm), is exactly equal to the equivalent physical diameter of a rigid sphere. Therefore, for cases when *a* is greater than the physical cell dimensions, the cell complexity enhances performance, since it leads to an increase in *R*. On the other hand, if *a* is smaller than the physical size of the object being filtered, then the cell complexity (shape or flexibility) limits performance. A comparison between the virtual and physical dimensions of algae is presented in Table 1.

The virtual size of the M1 cell is larger than its physical size, which implies that the cell complexity actually enhances the filtration performance. A possible explanation for this behavior is that the cell roughness induces additional lift forces, but we have not performed experiments to confirm this behavior.

**Figure 12:**
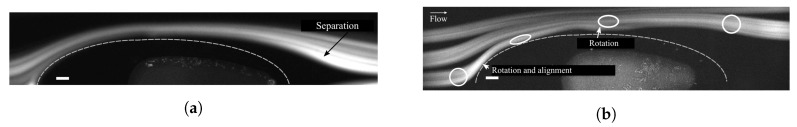
Streaklines showing trajectories of naturally fluorescent algae around a trilobite filtration unit: clogging is prevented by controlling the flow fields. (**a**) Hydrodynamic interactions are utilized to lift the disk-like F2 cell away from the filter blades and into the bulk flow. (**b**) Filtering of a sphere-like cell without signs of clogging: The external flow is from left to right. Scale bar is 50 μm.

The value of *a* reported for the sphere-like M2 cell is smaller than its physical size and means that the cell complexity is performance limiting. The reason for this behavior is that low-velocity rolling over the filtration pillars, which is used to handle rigid spheres of the same size, cannot be used for filtration of the M2 cell as these types of interactions were found to clog the device. Instead, the interactions with filtration structures must be minimized, which is achieved by utilizing inertial effects to induce lateral migration across streamlines, as seen in the visualization in Figure 12a. This clog-free mitigation mechanism comes at the expense of a slight decrease in concentration performance, since the amount of filtrate fluid is reduced relative to the feed fluid (the extent of the filtrate layer is reduced).

The virtual diameter of the rod-like F1 cell is *a* = 22 μm, which is comparable to its smallest dimension, i.e., the width (*w* = 19 μm). This similarity implies that the filtration mechanism is dominated by the smallest dimension of the cell, and this is due to alignment with the upstream body of the trilobite. The alignment results from a strong pressure gradient, which pushes the cell against the trilobite structure.

Finally, the performance for filtering disk-shaped F2 cells is also governed by the smallest cell dimension. This is directly seen as the width, *w*, and the virtual dimension, *a*, are almost identical (Table 1). Again, it is alignment with the upstream trilobite body that causes similarity between the cell width and the virtual size, and this is because the cell is pushed against it by a stagnation flow. This is clearly seen in the pathline representation in Figure 12b, showing the the trajectory of this cell around a filtration unit. However, also note how the cell diverts from the flow and is lifted into the bulk. This diversion is caused by a rotation near the first filter blades, which is the result of an adverse pressure gradient. At this point, the main part of the cell faces the flow, causing a strong lift force to push it farther away from the filter blades and into the bulk flow.

**Figure 13:**
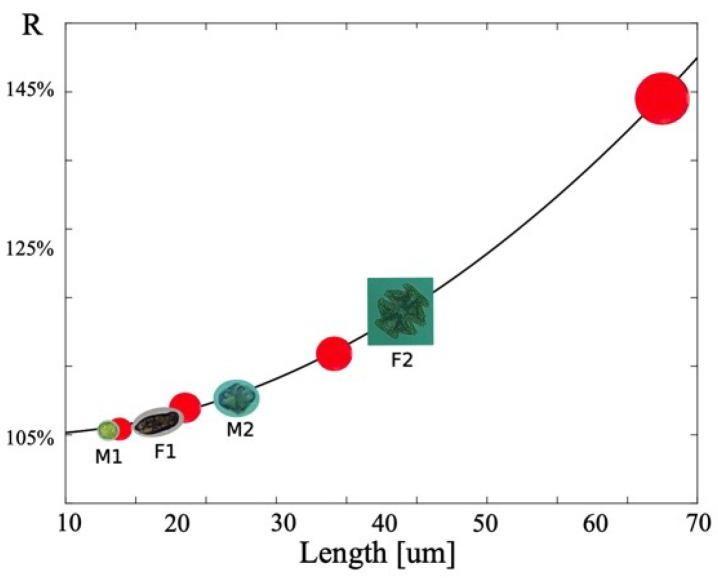
Graphical presentation of the concentration ratio, R, versus particle and cell size: based on the smallest dimension, e.g., the thickness of disks and the diameter of rods, the device shows similar concentration performance when applied to cells as when applied to synthetic beads. This similarity is convenient as the device can be calibrated with beads for real-life applications.

Figure 13 is a plot of the maximum achievable *R* without clogging. The red dots are the data points obtained using filtration particles (21, 24, 32, and 69 μm), and the solid line is a second-order fit to the data points ($r^{2}$ = 0.9997), achieved by least square fitting. The concentration ratios *R* of algae are indicated by images and is plotted against their smallest dimension, *w*. The graph demonstrates that the device performance for cells, when judged by *w*, is comparable to the device performance for treating rigid spheres. This similarity is convenient as the filter can be calibrated using test particles.

## 8. Conclusions

In the trilobite filtration method reviewed here, inertial flow fields are utilized to avoid clogging of the filter structures by minimizing the degree of direct particle interactions. We have previously described [20,21] how these favorable flow fields can be tuned to mitigate clogging issue for sized-based filtration of synthetic beads and for separation of live cells with complexity in shape, size, and deformability.

Here, we devote special attention to the modular flow setup and we describe the optical setup enabling flow and particle velocities in meters per second to be measured by PTV and μPIV. A detailed description of crucial calibration and validation procedures is provided. Especially, a comparison of our PIV data to a known flow profile is presented for the first time. The experimental procedures are explained in sufficient detail to make replication feasible, and the recommendations provided can serve as guidelines for building and testing tunable microfluidic setups beyond trilobite filtration.

In the last part of the paper, we compare the device performance of rigid spheres to that of cells. By introducing a virtual diameter for cells, we deconvolute influences of shape from size on the filtration mechanism. We find that the cell shape has only a weak influence on the device performance and that it is the smallest cell dimension that dictates the concentration ratio. Importantly, we find that the flow fields used to handle rigid spheres can be used for filtering complex cells without clogging. This is convenient as the device can be calibrated using rigid, well-defined spheres.

## Figures and Tables

**Table 1:** Results of algae separation experiments used to assess the influence of cell complexity on the filtration performance: here, *w* is the cell smallest dimension, *L* is cell largest dimension, and *a* is the virtual diameter. The M1 and M2 cells are almost spherical, while the F1 and F2 cells are rod and disk-shaped, respectively.

Name	Shape	*R*	*w* [μm]	*l* [μm]	*a* [μm]
M1	Sphere	103%	13	17	22
M2	Sphere	111%	29	30	26
F1	Rod	106%	19	41	22
F2	Disk	121%	37	77	38
